# Temporal trends of skin and soft tissue infections caused by methicillin-resistant *Staphylococcus aureus* in Gabon

**DOI:** 10.1186/s13756-024-01426-0

**Published:** 2024-06-25

**Authors:** Christiane Sidonie Gouleu, Maradona Agbanrin Daouda, Sam O’neilla Oye Bingono, Matthew Benjamin Bransby McCall, Abraham Sunday Alabi, Ayola Akim Adegnika, Frieder Schaumburg, Tobias Grebe

**Affiliations:** 1 Centre de Recherches Médicales de Lambaréné, Lambaréné, Gabon; 2 Radboud Center for Infectious Diseases, Department of Medical Microbiology, Radboud University Medical Center, Nijmegen, Netherlands; 3 Institute of Tropical Medicine, Eberhard Karls University of Tübingen, Tübingen, Germany; 4 Institute of Medical Microbiology, University Hospital Münster, Münster, Germany

**Keywords:** Soft tissue infections, Methicillin-resistant *Staphylococcus aureus*, Sub-Saharan Africa, Low and middle income countries, Cross sectional studies

## Abstract

**Background:**

Methicillin-resistant *Staphylococcus aureus* (MRSA) is one of the leading causes of mortality due to bacterial antimicrobial resistance. While *S. aureus* is common in skin and soft tissue infections (SSTI) in Africa, data on MRSA rates are scarce and reports vary widely across the continent (5%-80%). In this study, we describe the proportion of MRSA causing SSTI in Lambaréné, Gabon, over an 11-year period.

**Methods:**

We retrospectively analyzed data from 953 bacterial specimens collected from inpatients and outpatients with SSTI at the Albert Schweitzer Hospital, Lambaréné, Gabon, between 2009 and 2019. We determined temporal changes in the prevalence of MRSA and identified risk factors for SSTI with MRSA.

**Results:**

68% of all specimens with bacterial growth yielded *S. aureus* (n = 499/731), of which 7% (36/497) with antimicrobial susceptibility testing were identified as MRSA. Age above 18 years, admission to the surgical ward, and deep-seated infections were significantly associated with MRSA as the causative agent. After an initial decline from 7% in 2009, there was a marked increase in the proportion of MRSA among all *S. aureus* from SSTI from 3 to 20% between 2012 and 2019. The resistance rate to erythromycin was significantly higher in MRSA than in methicillin-susceptible *S. aureus* (73% vs. 10%), and clindamycin resistance was detected exclusively in MRSA isolates (8%).

**Conclusion:**

The increasing proportion of MRSA causing SSTI over the 11-year period contrasts with many European countries where MRSA is on decline. Continuous surveillance of MRSA lineages in the hospital and community along with antibiotic stewardship programs could address the increasing trend of MRSA.

**Supplementary Information:**

The online version contains supplementary material available at 10.1186/s13756-024-01426-0.

## Background

*Staphylococcus aureus* is a Gram-positive bacterium that is part of the normal human flora and persistently colonizes about 30% of the human population [1]. However, under favorable conditions, including breaching the skin barrier or in hosts with an immature or compromised immune system, *S. aureus* is capable of causing a wide spectrum of infections [2]. These include skin and soft tissue infections (SSTI), bone and joint infections, pneumonia, endocarditis and sepsis [3].

According to the recent Global Burden of Disease Study 2019, *S. aureus* was the leading bacterial pathogen, accounting for more than one million deaths worldwide [4], while methicillin-resistant *S. aureus* (MRSA) was the second leading cause of death worldwide due to antimicrobial resistance [5].

The worldwide proportion of MRSA among *S. aureus* causing infections varies widely even within countries or regions on different continents. Reported frequencies range from less than 5% in northern Europe to up to 50% in southern Europe, around 50% in the United States, and 56–86% in parts of East Asia [6, 7]. Data from the African continent is mostly available for the Mediterranean region (19–52%), Nigeria (11–31%), and South Africa (24%), but scarce and primarily obtained from single-center studies for other Sub-Saharan countries [8].

In Africa, the high rates of SSTI are closely linked to the prevalence of *S. aureus*, which is fueled by the tropical climate and high rate of scabies and other skin diseases [9, 10]. It is estimated that the prevalence of MRSA-associated SSTI in Africa may exceed 50% [11–13]. In developing countries, such as Gabon, antibiotic resistance poses a major public health threat, but studies investigating the prevalence of antimicrobial resistance are limited [14, 15]. Data on temporal changes in the proportion of MRSA (among all *S. aureus*) causing SSTI in Gabon are currently not available. This cross-sectional study aimed to evaluate the prevalence of MRSA causing SSTI during 2009–2019 in a secondary care hospital in Lambaréné, Gabon.

## Methods

### Study design

We performed a historical cross-sectional study at the Albert Schweitzer Hospital (ASH) in Lambaréné, Gabon. The ASH has about 150 beds and is annually used by approximately 50,000 people. It offers services including surgery, pediatrics, internal medicine, gynecology, and emergency medicine. Between 2009 and 2019, all cases of SSTI with a specimen sent to the microbiology laboratory were screened for inclusion. From a total of 953 cases, 836 cases were included after excluding duplicate specimen from a patient’s current episode of infection. SSTI were stratified into superficial (impetigo, ecthyma, folliculitis, cellulitis, erysipelas, superficial abscess or wound infections) and deep-seated infections (subcutaneous abscess, pyomyositis, necrotizing fasciitis) as previously described [16].

Socio-demographic and clinical data were collected from the microbiology laboratory and medical records. Patients below 18 years were considered as children. Outpatients and non-admitted patients from other hospital settings were considered as from the community. Empirical antimicrobial therapy was defined as prescription of antimicrobial agents for the patient’s current episode of infection prior to specimen collection or pending microbiological diagnostics. Empirical therapy and the number of previously prescribed antibiotics were obtained from the laboratory request form.

### Culture and identification

Samples from SSTI (e.g., swabs, pus) were cultured on Columbia blood agar (Oxoid, Basingstoke, Hampshire, United Kingdom) for 24–48 h at ambient air. Colonies suggestive for *S. aureus* were tested for catalase (BD BBL Catalase, Becton Dickinson, Franklin Lakes, NJ, USA), coagulase (BD BBL Coagulase Plasma, BD) and a slide agglutination test (Pastorex Staph Plus, Bio-Rad, Hercules, France) according to the manufacturer´s instructions.

### MRSA identification and antimicrobial susceptibility testing

Antimicrobial susceptibility testing was performed using disc diffusion test according to the Clinical Laboratory Standards Institute (CLSI) guidelines and inhibition zone diameters were interpreted with CLSI breakpoints [17]. Intermediate results were considered non-susceptible along with the resistant isolates in this study.

MRSA was screened using cefoxitin disks (30 µg, Oxoid, USA) and methicillin resistance was confirmed by the detection of the Penicillin-binding Protein-2a (PBP2a) using a slide latex agglutination assay (Oxoid) following the manufacturer’s instruction.

As part of a clinical trial requirement, the microbiology laboratory successfully participates in regular external quality assurance (EQA) programs, addressing species identification and susceptibility testing. The EQA is part of the WHO/NICD Proficiency Testing Scheme and is organized by the Contract Laboratory Services (CLS), Johannesburg, South Africa.

### Data analysis

All data analyses were performed using the software ‘‘R’’ (http://cran.r-project.org, Version: 4.3.1). Pearson’s chi-square test or Fisher’s exact test were used to analyze categorical data, t-test to analyze continuous data. Due to the retrospective nature of our data collection, we were unable to obtain certain missing patient information. Therefore, we report both absolute numbers and percentages of observed events, calculated based on the total number of events for which data were available.

## Results

Out of 836 non-duplicate specimens obtained from SSTI between 2009 and 2019, 87.4% (731/836) yielded bacterial growth of which 68.3% (499/731) were identified as *S. aureus*. The median age of SSTI patients with a positive culture was 6 (0–89) years and the majority of cases were in children (432/678). Infection with *S. aureus* was significantly associated with young age, highlighted by *S. aureus* constituting the causative agent in 77% (334/432) of SSTI cases in children (OR 2.9, 95% CI 2.1–4.1, *p* < 0.001). SSTI with *S. aureus* was more prevalent in patients without empirical antimicrobial therapy (70%, 301/429), and previous prescription of more than two antibiotics was associated with a decrease in *S. aureus* SSTI (43%, 9/21; OR 0.3, 95% CI 0.1–0.8, *p* = 0.013). More than half of the cases (54%, 268/499) were categorized as community-acquired.

**Fig. 1:**
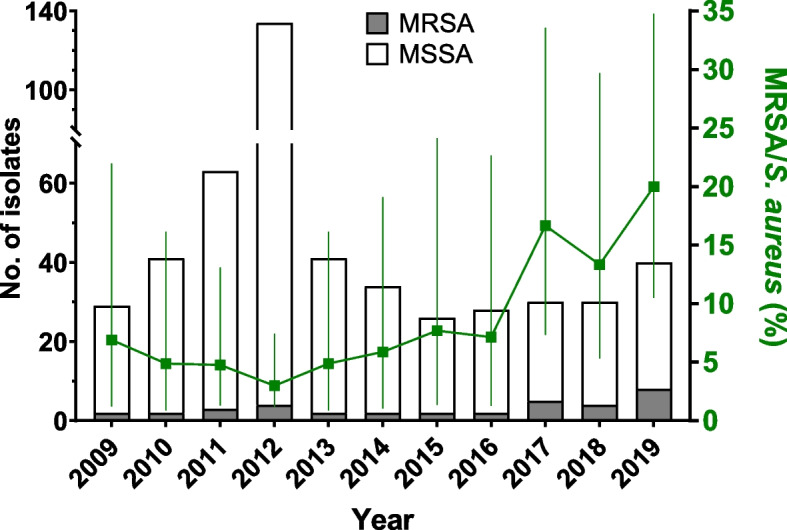
Temporal trends of the proportion of methicillin-resistant *Staphylococcus aureus* (MRSA) in *S. aureus* skin and soft tissue infection (SSTI) samples from 2009 to 2019. Stacked bars show the number of MRSA (grey) or methicillin-susceptible *S. aureus* (MSSA, white) isolates per year. Green line with error bars shows the proportion of MRSA among *S. aureus* (as percentage with 95% confidence interval) for each year. It should be noted that the peak in the total number of *S. aureus* from SSTI in 2011 and 2012 may be attributed to an increased number of detected SSTI cases among participants in a malaria vaccine trial conducted at the study site between 2009 and 2013

Two of the 499 identified *S. aureus* isolates were excluded from subsequent analyses due to a lack of antimicrobial susceptibility testing. This reduced the number of isolates to 497. The overall proportion of MRSA among all *S. aureus* isolates collected during the study period was 7.2% (36/497). The proportion of MRSA in SSTI initially declined from 7 to 3% between 2009 in 2012, followed by a marked increase to 20% in 2019 (Fig. 1).

Methicillin resistance rates were four times higher in isolates from adults than children (16% vs. 4%, *p* < 0.001) with a median age of patients with MRSA-associated SSTI of 24 (range: 0–76) years (Table 1).

**Table 1:** Demographic characteristics and clinical data of patients with SSTI caused by *S. aureus*

	**Total *N* = 497**	**MRSA *N* = 36**	**MSSA *N* = 461**	**OR [95% CI]**	***p* value**
**Age** (median [range])	4 [0;84]	24 [0;76]	3 [0;84]	1.03 [1.05;1.02]	** < 0.001**
**Age group**					
Children	334 (72%)	13 (3.9%)	321 (96.1%)	Ref	Ref
Adults	130 (28.0%)	21 (16.2%)	109 (83.8%)	4.72 [2.30;10.0]	** < 0.001**
**Sex**					
Male	257 (53.9%)	20 (7.8%)	237 (92.2%)	Ref	Ref
Female	220 (46.1%)	15 (6.8%)	205 (93.2%)	0.87 [0.42;1.74]	0.82
**Clinical sample**					
Superficial	473 (95.2%)	28 (5.9%)	445 (94.1%)	Ref	Ref
Deep-seated	24 (4.8%)	8 (33.3%)	16 (66.7%)	7.94 [2.96;19.9]	** < 0.001**
**Ward**					
Out-patient department	267 (53.7%)	14 (5.2%)	253 (94.8%)	Ref	Ref
Other	23 (4.6%)	3 (13.0%)	20 (87.0%)	2.79 [0.58;9.62]	0.18
Pediatric	120 (24.1%)	3 (2.5%)	117 (97.5%)	0.48 [0.10;1.53]	0.23
Surgery	87 (17.5%)	16 (18.4%)	71 (81.6%)	4.05 [1.87;8.85]	** < 0.001**
**Empiric antimicrobial therapy**					
No	299 (60.2%)	20 (6.7%)	279 (93.3%)	Ref	Ref
Yes	200 (39.8%)	16 (8.1%)	182 (92.0%)	1.21 [0.60;2.39]	0.72
**Number of previously prescribed antibiotics**					
None	299 (60.2%)	20 (6.7%)	279 (93.3%)	Ref	Ref
1	156 (31.4%)	10 (6.4%)	146 (93.6%)	0.96 [0.42;2.08]	0.93
2	33 (6.6%)	4 (12.1%)	29 (87.9%)	1.97 [0.53;5.71]	0.28
> 2	9 (1.8%)	2 (22.2%)	7 (77.8%)	4.15 [0.53;19.2]	0.15

We found that patient age above 18 years (OR 4.7, 95% CI 2.3–10.0, *p* < 0.001), admission to the surgical ward (OR 4.1, 95% CI 1.9–8.9, *p* < 0.001), and deep-seated infections (OR 7.9, 95% CI 3.0–19.9, *p* < 0.001) were significantly associated with MRSA as the causative agent (Table 1). We observed that the odds of infection with MRSA increased with the number of previously prescribed antibiotics. However, the association was not statistically significant (Table 1).

The rate of resistance to erythromycin (73% vs. 10%) and gentamicin (15% vs. 3%) was significantly higher in MRSA than in MSSA isolates, and clindamycin resistance was detected exclusively in one MRSA isolate (8%, Table 2). Temporal trends in the antimicrobial resistance profile of MRSA and MSSA are provided in Figure S1.

**Table 2:** Antibiotic resistance of *S. aureus* isolates

	**Number of resistant isolates**^**a**^				
	**MRSA**		**MSSA**		***p* value**
	**[% (95% CI)]**	**[n**_**resistant**_**/n**_**tested**_**]**	**[% (95% CI)]**	**[n**_**resistant**_**/n**_**tested**_**]**	
Penicillin	100% (88–100%)	36/36	92% (89–94%)	409/445	0.10
Gentamicin	15% (3–46%)	2/13	3% (1–6%)	7/266	**0.06**
Ciprofloxacin	27% (7–61%)	3/11	7% (2–18%)	4/57	0.078
Clindamycin	8% (0–40%)	1/12	0% (0–2%)	0/273	**0.04**
Erythromycin	73% (54–86%)	24/33	10% (8–14%)	44/427	** < 0.001**
Tetracycline	43% (23–66%)	9/21	52% (46–58%)	132/253	0.55
Cotrimoxazole	14% (4–37%)	3/21	7% (4–12%)	12/182	0.19

^**a**^Not all isolates included in the study were tested for all antibiotics

## Discussion

We performed a historical cross-sectional study on *S. aureus* SSTI and found an increase in the proportion of MRSA over a period of 11 years.

The majority of SSTI are caused by *S. aureus*. In our study, 68% (499/731) of all culture-positive SSTI samples yielded *S. aureus* as the causative agent, which is consistent with findings from Gabon (75%), Kenya (46%) or Switzerland (65%) [18–20].

The overall proportion of MRSA among all *S. aureus* in our study was 7%, while the proportion ranged from 3–8% between 2009 and 2016, followed by a marked increase to 20% in 2019. In line with this, previous studies conducted between 2008 and 2012 in Lambaréné observed MRSA rates of 6–11% [14, 20]. Similarly, a prospective multi-center study conducted between 2016 and 2018 in Gabon found a rate of 16% MRSA among all *S. aureus* isolates [15]. While the above studies included isolates from all types of infections, a study conducted in several Sub-Saharan African countries, including Gabon, on the management of *S. aureus* SSTI detected 6.5% MRSA [16]. In this multicenter study, the rate of MRSA was similar in superficial and deep-seated infections which is divergent to our finding of significantly higher MRSA rates in deep-seated compared to superficial infections.

The observed increase in the rate of MRSA in isolates originating from the surgical ward of the hospital (18%) may be attributed to the fact that MRSA patients are more likely to fail empirical antimicrobial therapy and therefore progress to more severe infections requiring surgical intervention. This is supported by the high proportion of MRSA-associated purulent infections in our study, such as abscesses, that require surgical drainage [16]. Alternatively, it might point toward a nosocomial transmission of MRSA on surgical wards. Such a transmission might be further supported by a lower MRSA prevalence on pediatric wards. However, such a nosocomial source has not been investigated yet. Past studies rather suggest multiple sources for MRSA colonization as indicated by *S. aureus* protein A (*spa)*-types (t186, t653, t121, t729) or multilocus sequence types (ST5, ST8, ST88) among five MRSA isolates from health care workers, patients and the general population in Lambaréné, Gabon [21]. Noteworthy, these sequence types are also among the major MRSA lineages in Africa [22]. Further updated genotyping analysis of MRSA in our study setting are warranted to identify potential new sources and transmission chains.

A recent survey conducted among healthcare workers in our study area revealed a lack of knowledge on causes for the emergence of antimicrobial resistance and poor antibiotic stewardship [23]. Irrational prescription habits and easy accessibility to antimicrobials without a prescription might underlie the increase in MRSA shown in our study region.

A peak in the incidence of *S. aureus* SSTI was observed in 2012, while the proportion of MRSA was very low (Fig. 1). A malaria vaccination trial was conducted at our study site from 2009 to 2013 [24]. During the primary vaccination series and follow-up visits, the participants were seen by a physician which may have increased the chance of detecting SSTI that were previously undetected, thus, affecting sample sizes during this period. However, there was no active screening for SSTIs, and specimens were only collected and sent to the microbiology laboratory if there was clinical evidence of an SSTI. There were two age groups in the study, one comprising infants aged 6–12 weeks and the other comprising young infants aged 5–17 months. This may account for the observed proportion of approximately two-thirds of children among SSTI patients in our study and the median age of 4 years among patients with *S. aureus* SSTI. However, previous studies in Sub-Saharan Africa have shown that the median age of patients with *S. aureus* infections, including SSTI, can be as low as 3–4 years [12, 16].

Our study has several limitations. First, since this is a historical cross-sectional study, we exclusively relied on the medical and laboratory records of the patients for whom microbiological testing was requested. Thus, patients who have not undergone microbiological examination could have been missed in our study, possibly underestimating the rate of SSTI in the study area. Due to the limited medical data, the duration of stay, or applied treatment and outcome of the patients was not completely assessed. In addition, we were unable to classify infections according to standard definitions of hospital-acquired, community-acquired, or healthcare-associated infections [25]. The availability of these data would help to elucidate the role of iatrogenic infections, including those associated with healthcare interactions but emerging within the community. Second, we were not able to analyze the genotype of the MRSA isolates regarding the sequence type or clonal complex attribution. Lastly, the inclusion of a single hospital may not be representative of the entire country or Central African region.

## Conclusion

In conclusion, the proportion of MRSA among *S. aureus* SSTI increase steadily over an 11-year period. Continuous surveillance of MRSA lineages in the hospital and community along with antibiotic stewardship programs could address the increasing trend of MRSA in Lambaréné.

### Supplementary Information

Supplementary Material 1.

## Data Availability

The datasets used and/or analyzed during the current study are available from the corresponding author on reasonable request.

## Abbreviations

SSTI: Skin and soft tissue infections
MRSA: Methicillin-resistant Staphylococcus aureus
MSSA: Methicillin-susceptible Staphylococcus aureus
OR: Odds ratio
95% CI: 95% Confidence interval
