# Supplementary material for: Temporal trends of skin and soft tissue infections caused by methicillin-resistant Staphylococcus aureus in Gabon
Source: Antimicrob Resist Infect Control. 2024 Jun 25;13:68. doi: 10.1186/s13756-024-01426-0 (PMC11201302; doi:10.1186/s13756-024-01426-0)
Supplement: Supplementary file 1 — Supplementary Material 1. [file 13756_2024_1426_MOESM1_ESM.docx]

Supplementary materials for

**Temporal trends of skin and soft tissue infections caused by methicillin-resistant *Staphylococcus aureus* in Gabon**

**Authors:**

Christiane Sidonie Gouleu^1^, Maradona Agbanrin Daouda^1^, Sam O’neilla Oye Bingono^1^, Matthew Benjamin Bransby McCall^2^, Abraham Sunday Alabi^1^, Ayola Akim Adegnika^1,3^, Frieder Schaumburg^1,4^, Tobias Grebe^4^


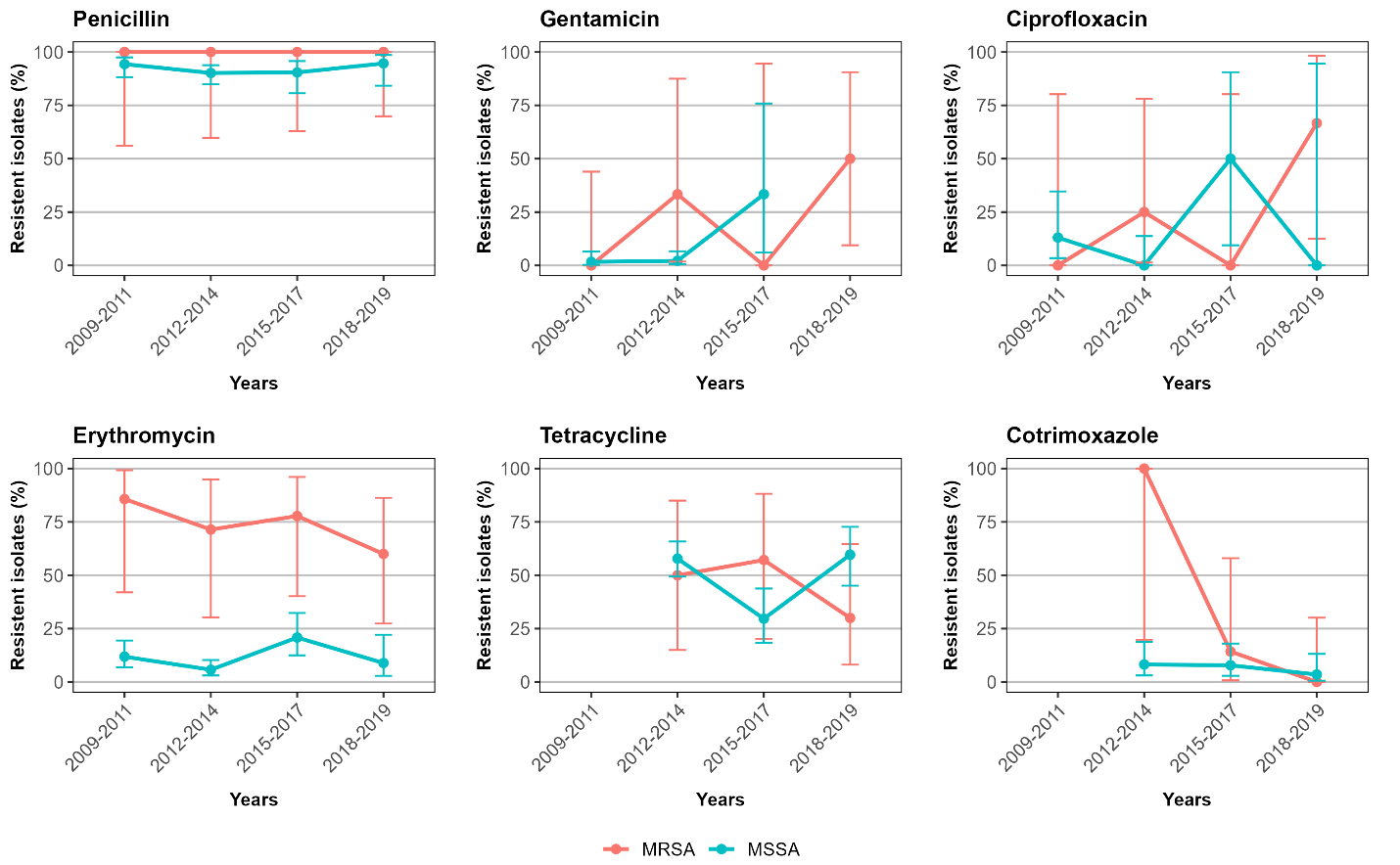


**Figure S1.** **Temporal trends in the antimicrobial resistance profile of MRSA and MSSA.** Proportion of methicillin-resistant *Staphylococcus aureus* (MRSA) and methicillin-susceptible *Staphylococcus aureus* (MSSA) isolates resistant to each antimicrobial agent during the study period. The time frame of the study was divided into four sections of 2-3 years to account for the low sample number in some individual years. Clindamycin was excluded because there was only one resistant isolate in our study. It should be noted that in some years sample numbers were low for some antibiotics, so proportions should be interpreted with caution (see Table 2). Error bars represent 95% confidence intervals. Prior to 2012, susceptibility testing for tetracycline and cotrimoxazole was not performed.
